# Reactive astrocytes in multiple sclerosis impair neuronal outgrowth through TRPM7‐mediated chondroitin sulfate proteoglycan production

**DOI:** 10.1002/glia.23526

**Published:** 2018-11-19

**Authors:** Alwin Kamermans, Kirsten E. Planting, Kees Jalink, Jack van Horssen, Helga E. de Vries

**Affiliations:** ^1^ Department of Molecular Cell Biology and Immunology, Amsterdam Neuroscience, MS center Amsterdam, Amsterdam UMC Vrije Universiteit Amsterdam Amsterdam The Netherlands; ^2^ Department of Cell Biology The Netherlands Cancer Institute Amsterdam the Netherlands

**Keywords:** astrocytes, chondroitin sulfate proteoglycan, multiple sclerosis, neurodegeneration, TRPM7

## Abstract

Multiple sclerosis (MS) is a chronic inflammatory disorder of the central nervous system (CNS), characterized by inflammation‐mediated demyelination, axonal injury and neurodegeneration. The mechanisms underlying impaired neuronal function are not fully understood, but evidence is accumulating that the presence of the gliotic scar produced by reactive astrocytes play a critical role in these detrimental processes. Here, we identified astrocytic Transient Receptor Potential cation channel, subfamily M, member 7 (TRPM7), a Ca^2+^‐permeable nonselective cation channel, as a novel player in the formation of a gliotic scar. TRPM7 was found to be highly expressed in reactive astrocytes within well‐characterized MS lesions and upregulated in primary astrocytes under chronic inflammatory conditions. TRPM7 overexpressing astrocytes impaired neuronal outgrowth in vitro by increasing the production of chondroitin sulfate proteoglycans, a key component of the gliotic scar. These findings indicate that astrocytic TRPM7 is a critical regulator of the formation of a gliotic scar and provide a novel mechanism by which reactive astrocytes affect neuronal outgrowth.

## 1 INTRODUCTION

Multiple sclerosis (MS) is a progressive inflammatory and demyelinating disease of the central nervous system (CNS). It is estimated that more than 2 million people worldwide suffer from MS, making it one of the most common chronic neurological disease of young adults. MS is generally considered as an auto‐immune disease in which autoreactive T‐lymphocytes and infiltrating monocytes induce focal inflammation and demyelination. Although demyelination is the most prominent histopathological feature of MS lesions, axonal damage has been shown to be a more accurate predictor of long‐term clinical outcome and disease progression (Lubetzki & Stankoff, 2014).

In the last decades, great progress has been made in understanding and controlling the inflammatory components of MS, but the pathophysiological mechanisms that contribute to neurodegeneration and hamper neuroregeneration remain largely elusive (Lassmann, van Horssen, & Mahad, 2012). Astrocytes play an essential role in the regulation of CNS homeostasis by supporting neuronal function and metabolism (Finsterwald, Magistretti, & Lengacher, 2015). In MS lesions, astrocytes gain a reactive phenotype due to dysfunction of the blood–brain barrier, ongoing inflammation and chronic demyelination. Reactive astrocytes can facilitate blood–brain barrier repair, secrete immunosuppressive molecules and exert neuroprotective properties. However, they can also exacerbate inflammation and blood–brain barrier leakage by secreting proinflammatory molecules, thereby facilitating immune cell influx (Mizee et al., 2014; Sofroniew & Vinters, 2010; van Doorn et al., 2012). Furthermore, reactive astrocytes generate a glial scar, marked by interwoven astrocytic processes accompanied by an excessive accumulation of extracellular matrix (ECM) components. This glial scar may form a physical barrier around areas of demyelination to prevent widespread tissue damage, but also inhibits remyelination and axonal outgrowth (Nair, Frederick, & Miller, 2008; Sofroniew & Vinters, 2010). Thus, reactive astrocytes play a dual role in the pathogenesis of MS (for review see [Brosnan & Raine, 2013; Miljković, Timotijević, & Mostarica Stojković, 2011]).

Recent evidence shows that phospholipase C (PLC), which subsequently activates the inositol 1,4,5‐trisphosphate (IP_3_)‐Ca^2+^ signaling cascade, is involved in pathophysiological functions of astrocytes (Kanemaru et al., 2013). It has been suggested that G protein‐coupled receptors, which can be activated by a wide array of ligands, are primarily responsible for mediating (anti)inflammatory responses via PLC. Therefore, a better understanding of their downstream signaling pathways may lead to the development of novel therapeutics, aimed to dampen the inflammatory responses in astrocytes. In this regard, altered expression and/or activation of IP_3_‐dependent ion channels on astrocytes may play an important role in MS pathogenesis. Within this class of ion channels, transient receptor potential (TRP) channels have been recognized to be involved in numerous pathological conditions, including neurological disorders (Jordt & Ehrlich, 2007). Interestingly, previous reports identified Transient Receptor Potential Melastatin 7 (TRPM7), a member of the TRP channel family with inherent kinase activity, to be involved Alzheimer's disease, Parkinson disease, amyotrophic lateral sclerosis and stroke via both Ca^2+^ and Mg^2+^ dependent and independent mechanisms (Sun, Sukumaran, Schaar, & Singh, 2015). In addition, comparative gene expression analysis identified TRPM7 as one of the common upregulated genes in Alzheimer's disease and MS compared with healthy controls (Tseveleki et al., 2010). Besides a role for TRPM7 in neurodegenerative diseases, TRPM7 is involved in fibrotic diseases, such as liver fibrosis, pulmonary fibrosis, and cardiovascular fibrosis (Du et al., 2010; Fang et al., 2013; Yu et al., 2013). These disorders are characterized by excessive accumulation of ECM, similar as seen in MS lesions (Lau, Cua, Keough, Haylock‐Jacobs, & Yong, 2013; van Horssen, Bö, Dijkstra, & de Vries, 2006; Van Horssen, Dijkstra, & De Vries, 2007). Here, we provide evidence for increased expression of TRPM7 in astrocytes within MS lesions and show that astrocytic TRPM7 impairs neurite outgrowth by enhanced production of ECM proteins.

## 2 MATERIALS AND METHODS

### 2.1 Brain tissue

Brain samples were obtained from 9 MS patients and 4 nonneurological controls, in collaboration with the MS Centrum Amsterdam and the Netherlands Brain Bank. Detailed clinical data are summarized in Table 1. All donors, or their next of kin, had given informed consent for brain autopsy and use of their brain material and clinical information for research purposes.

**Table 1:** Clinical data of MS patients and non‐neurological controls

Case	Age (years)	Type of MS	Gender	Post‐mortem delay (h:Min)	Disease duration (years)	Lesion stage	Cause of death
MS 1	69	SP	F	7:30	>15	CA	Respiratory failure with hearth failure
MS 2	66	SP	F	6:00	>15	CIA	Unknown
MS 3	77	RR	F	10:00	>15	CIA	Unknown
MS 4	51	SP	M	11:00	>15	A	Infection
MS 5	49	RR	M	8:00	>15	CA	Pneumonia
MS 6	41	PP	M	7:23	14	CA	Urosepsis and pneumonia
MS 7	49	PP	F	8:30	>15	CA	Legal euthanasia
Control 1	62		M	Unknown			Unknown
Control 2	73		M	6:00			Colon carcinoma with liver metastases
Control 3	77		F	2:55			Unknown
Control 4	82		F	5:10			Pneumonia by haemothorax
Control 5	62		M	9:35			Unknown
Control 6	56		F	9:15			Myocardial infarction

MS, multiple sclerosis; SP, secondary progressive MS; RR, relapse remitting MS; PP, primary progressive MS; ND, non‐determined; NA, non‐applicable; M, male; F, female; a, active lesions; CA, chronic active lesions; CIA, chronic inactive lesions.

### 2.2 Immunohistochemistry

Formalin‐fixed paraffin‐embedded tissue was stained as described previously (Mizee et al., 2014). In short, deparaffinization and antigen retrieval, sections were incubated overnight with appropriate antibodies (for details, see Table 2) and subsequently incubated with horseradish peroxidase EnVision kit (Dako, Denmark) followed by 3,3’diaminobenzidine‐tetrahydrochloride dihydrate (Dako, Denmark). All sections were counterstained with hematoxylin after which they were analyzed with a light microscope (AXIO Scope A1, Carl Zeiss, Germany). For cellular localization studies, sections were incubated overnight with appropriate antibodies followed by incubation with Alexa‐488‐labeled goat antimouse IgG1 (1:200; Molecular Probes, USA) and analyzed by confocal microscopy (Leica DMI 6000 SP8, Leica, Germany).

**Table 2:** Antibody details

Antigen	Dilution	Antibody type	Source
Chondroitin 4 Sulfate	1:400	IgM	Cosmobio, Japan
Chondroitin 4 Sulfate	1:200	IgG1	Millipore, Germany
GFAP	1:300	IgG1	Sigma‐Aldrich, USA
HLA‐DR (MHC‐II)	1:100	IgG2b	eBioscience, USA
MAP2	1:200	Polyclonal	Sigma‐Aldrich, USA
PLP	1:500	IgG2a	Serotec, USA
TRPM7	1:50	Polyclonal	Alomone, Israel
TRPM7	1:200	IgG1	Stressmarq, Canada

**Table 3:** Primer sequences

Target gene	Species	Forward primer	Reverse primer
TRPM7	Rat	TTG GGA GAG ATG TGG TTG CC	AAT CCT TCC AAC CGT GCC GT
GAPDH	Rat	AGG TTG TCT CCT GTG ACT TC	CTG TTG CTG TAG CCA TAT C
TRPM7	Human	TAG CCT TTA GCC ACT GGA C	GCA TCT TCT CCT AGA TTT GC
GAPDH	Human	CCA TGT TCG TCA TGG GTG TG	GGT GCT AAG CAG TTG GTG GTG

### 2.3 Cell treatments

Primary human cerebellar astrocytes (ScienCell, USA) were cultured in astrocyte medium (ScienCell, USA). Human astrocytoma cells (U373) were cultured in Dulbecco's modified Eagle's medium (DMEM)/F12 (Life Technologies, USA) containing 10% fetal calf serum (FCS; Life Technologies, USA), and penicillin/streptomycin (50 mg/ml; Life Technologies, USA) in 5% CO^2^ at 37°C. For treatments, cells were incubated with tumor necrosis factor alpha (TNFα 10 ng/ml; Peprotech, UK) and interferon gamma (IFN‐γ; 10 ng/ml Peprotech, UK), transforming growth factor beta 1 (TGF‐β1; 10 ng/ml, R&D Systems, USA), interleukin 1 alpha (IL‐1α; 3 ng/ml, Sigma‐Aldrich, USA) or Complement component 1q (C1q; 400 ng/ml, Sigma‐Aldrich, USA) for 24 hr.

### 2.4 Primary rat astrocytes

Mixed glia cells were isolated from 0‐ to 1‐day‐old postnatal rats of either sex of which brains were placed in cold dissection medium consisting of HBSS (Invitrogen, Carlsbad, CA, USA), 1 mM sodium pyruvate (Sigma‐Aldrich, USA), 0.1% w/v D‐glucose (Riedel‐de Haën, Germany) and 10 mM HEPES (Invitrogen, USA). Cortices were dissociated in DMEM (Invitrogen, USA), containing 2.5% trypsin (Difco Laboratories, USA) and 10 mg/ml DNAse 1 (Roche, Germany) at 37°C for 20 min. Dissociated tissue was passed through a 100 μm cell strainer and centrifuged at 500g for 5 min. After which the pallet was resuspended in plating medium consisting of DMEM containing pyruvate, glucose and glutamine (Invitrogen, USA), penicillin (100 U/ml), streptomycin (100 mg/ml) (Lonza, Switzerland) and 10% FCS (Invitrogen, USA). Cells were cultured for 7–10 days. Microglia was removed from the mixed glia cell culture using an orbital shaker at 230 rpm for 3 hr.

### 2.5 Primary rat neurons

Neurons were isolated from 0 to 1 days old postnatal rats of either sex of which brains were placed in cold dissection medium consisting of HBSS (Invitrogen, Carlsbad, CA), 1 mM sodium pyruvate (Sigma‐Aldrich, USA), 0.1% w/v D‐glucose (Riedel‐de Haën, Germany) and 10 mM HEPES (Invitrogen, USA). Cortices were dissociated in DMEM (Invitrogen, USA), containing 2.5% trypsin (Difco Laboratories, USA) and 10 mg/ml DNAse 1 (Roche, Germany) at 37°C for 20 min. Dissociated tissue was passed through a 100 μm cell strainer and centrifuged at 500 g for 5 min. After which the pallet was resuspended in plating medium consisting of DMEM containing pyruvate, glucose and glutamine (Invitrogen, USA), penicillin (100 U/ml, Lonza, Switzerland), streptomycin (100 mg/ml, Lonza, Switzerland) and 10% FCS (Invitrogen, USA). Finally, primary neurons were plated on top of a confluent astrocyte feeder layer consisting of U373 astrocytes. Non‐adhered cells were removed 2 hr after plating. Culturing of primary neurons was performed in culture medium consisting of DMEM (Invitrogen, USA); penicillin (100 U/ml, Lonza, Switzerland), streptomycin (100 mg/ml, Lonza, Switzerland) glutamic acid (25uM),1 mM sodium pyruvate and 10% FCS (Invitrogen, USA) for 12–14 days in a humidified incubator at 37°C and 5% CO^2^.

### 2.6 RNA isolation and real‐time quantitative PCR

Gene expression analysis was performed on sub‐confluent primary human astrocytes or U373 astrocytoma cells. Messenger RNA was isolated using Trizol (Invitrogen, USA) according to manufacturer's protocol. mRNA concentration and quality were measured using Nanodrop (Nanodrop Technologies, USA). cDNA syntheses was performed using the Reverse Transcription System kit (Promega, USA) following manufacturers guidelines. RT‐PCR was performed as described previously (García‐Vallejo et al., 2004). Primer sequences used are listed in supplementary Table 3.

### 2.7 Retroviral‐induced overexpression of TRPM7

Human astrocytoma cells (U373) stably overexpressing TRPM7 (TRPM7^+^ astrocytes) and empty vector control (Mock astrocytes) were generated by retroviral transductions. HEK293FT cells were cultured in DMEM containing 10% FCS, 1% penicillin/streptomycin (50 mg/ml; Life Technologies, USA) at 37°C in a 5% CO^2^ incubator. HEK293FT cells were transfected with calcium phosphate as a transfection reagent. Medium was refreshed 6 hr posttransfection. Supernatant containing virus was collected and virus was concentrated by centrifugation. U373 cells were cultured in DMEM/F12 (Life Technologies, USA) containing 10% FCS (Life Technologies, USA), and penicillin/streptomycin (50 mg/ml; Life Technologies, USA) in 5% CO^2^ at 37°C. Transduction was carried out 24 hours after seeding of cells, medium of U373 cells was replaced with virus containing supernatant. Virus supernatant was replaced with appropriate medium after 6 hr incubation. Transduced cells were selected using 300 mg/ml G418 (Invitrogen, USA). The effect of TRPM7 overexpression on cell viability and proliferation was assessed by CellTiter 96® Aqueous One Solution Cell Proliferation Assay (Promega, The Netherlands) according to manufacturer's instructions.

### 2.8 Intracellular Ca^2+^ recording

Ca^2+^ recordings were performed as described previously (Langeslag, Clark, Moolenaar, van Leeuwen, & Jalink, 2007; Visser et al., 2013). In short, cells were grown in 24 wells plate and incubated with Oregon Green 488 BAPTA‐1‐AM (Molecular Probes, USA) followed by further incubation in 2 ml DMEM F/12. The plate was mounted on an IX81 inverted epifluorescence microscope (Olympus, Japan). Recordings were made at 37°C in 5% CO^2^ and 80% humidity. Excitation of Oregon Green‐488 was performed at 480/25 nm and emission was detected between 502 and 538 nm. All Ca^2+^ recordings were normalized by setting the response to ionomycin (Sigma‐Aldrich, USA) at 100%.

### 2.9 Assessment of neuron morphology

Astrocyte‐neuron co‐cultures were fixed in 4% PFA for 15 min. Fixed cells were, blocked in PBS containing 0.1% Triton X‐100 and 10% goat serum for 30 min and incubated with primary antibody directed against microtubule‐associated protein 2 (1:200 mouse anti microtubule‐associated protein 2 [MAP2], Sigma‐Aldrich, USA) in blocking buffer overnight at °4C. Subsequently, samples were incubated for 1 hr with Alexa conjugated secondary antibody (goat antirabbit IgG‐Alexa 488, 1:200; Molecular Probes, USA). Coverslips were mounted in DAPI solution after washing. To evaluate morphology of the cultured neurons, Sholl analysis plug‐in of NIN ImageJ software was used.

### 2.10 Chondroitinase ABC treatment

U373 astrocytes were digested with chondroitinase ABC (ChABC) (Sigma‐Aldrich, USA) to remove the chondroitin/dermatan sulfate structures. A confluent monolayer of U373 astrocytes was treated with a Tris HCL (200 mM) / sodium acetate (200 mM) buffer containing 10 U/ml chABC for 4 hr at 37°C and 5% CO^2^.

### 2.11 Immunocytochemistry

U373 astrocytes were incubated overnight with antibodies directed against chondroitin sulfate proteoglycans (CSPGs). Intact chondroitin sulfate side‐chains were detected with the 2H6 antibody (1:200, clone 2H6, Cosmo Bio, Germany) and core proteins using the BE‐123 antibody (1:200, clone BE‐123, Millipore, Germany,) following chABC predigestion. Subsequently, samples were incubated for 1 hr with Alexa‐488‐conjugated secondary antibodies (goat antimouse IgG1‐Alexa 488, 1:200; Molecular Probes, USA). Coverslips were mounted in MOWIOL after washing. Fluorescence analysis was performed with a Leica DM6000 microscope (Leica Microsystems, Germany).

### 2.12 Dot blot analysis

Cells were washed with ice‐cold PBS and lysed in 1 × sodium dodecyl sulfate (SDS) sample buffer (100 mM Tris–HCl pH 6.8, 4% SDS, 20% glycerol and 5% β‐mercaptoethanol). All samples were heated to 95°C for 5 min. The protein concentration of HDL3 was determined using the bicinchoninic acid assay (Thermo Scientific, Belgium), with BSA as the standard. An 1 μl containing 1 μg/μl protein was transferred onto a nitrocellulose membrane (Bio‐Rad, USA), set to dry and incubated overnight with 2H6 antibody (1:1,000, clone 2H6, Cosmo Bio, Germany) in Odyssey blocking buffer (LI‐COR, USA) diluted 1:1 in PBS, after initial blocking with blocking buffer for 1 hr at RT. Primary antibodies were detected by incubation with to corresponding IRDye secondary antibodies for 1 hr at RT in blocking buffer and the Odyssey infrared imaging system (LI‐COR, USA). Intensity measurements of CSPG immunoreactivity were obtained using ImageJ software.

## 3 RESULTS

### 3.1 Increased astrocytic TRPM7 expression in MS lesions

First, we investigated the cellular expression of TRMP7 in control brain tissue and well‐characterized MS lesions. White matter lesions were identified and classified by the absence of myelin (proteolipid protein [PLP]) and the presence of MHC Class II^+^ cells as reported before (Van Der Valk & De Groot, 2000).

**Figure 1:**
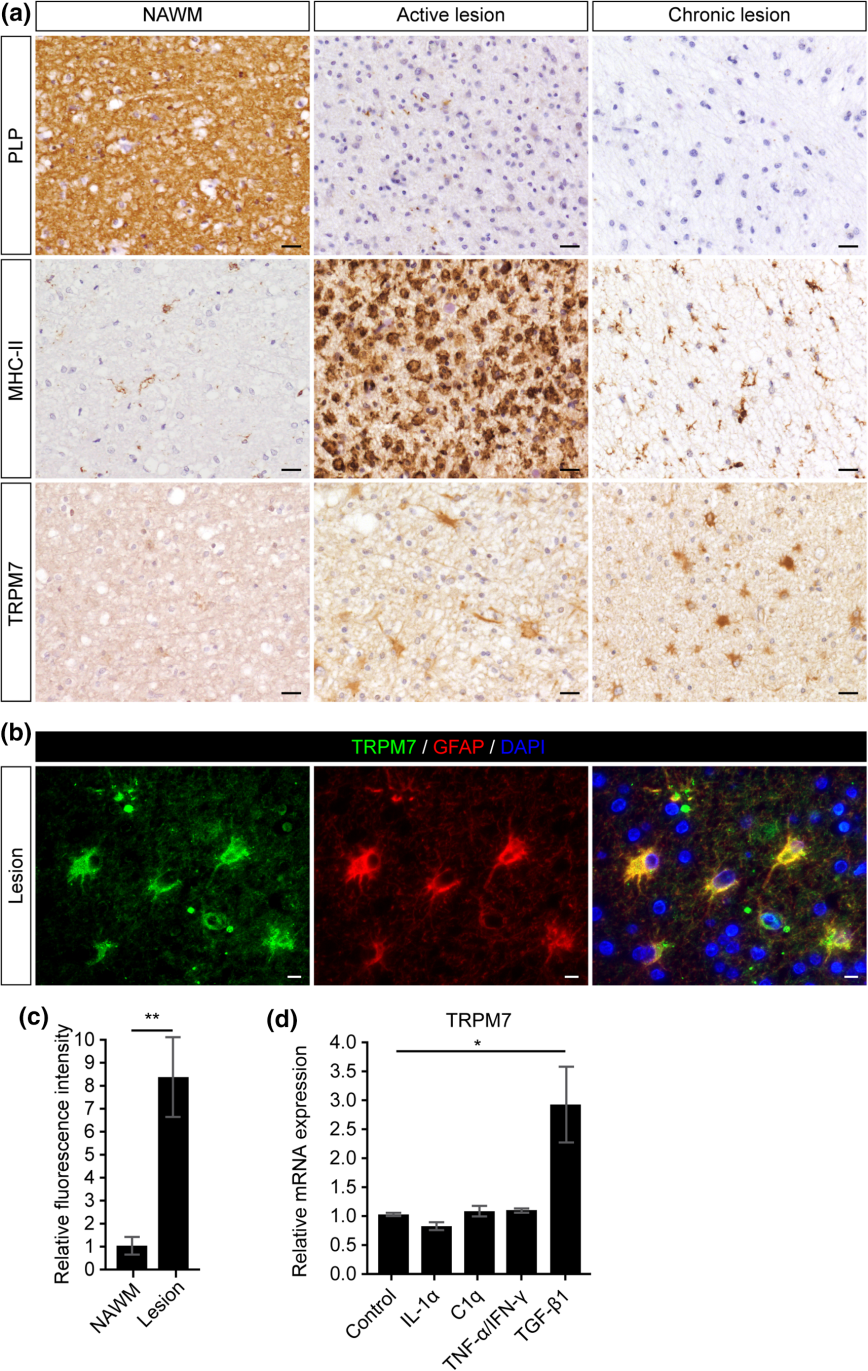
Increased astrocytic expression of TRPM7 in MS lesions. (a) MS lesions are characterized by loss of myelin (PLP) and abundant HLA‐DR (MHC‐II)‐positive cells. TRPM7 expression is markedly increased in active and chronic lesions particularly in cells with an astrocyte morphology (scale bar = 25 μm). (b) Double immunofluorescence labeling shows co‐localization of TRPM7 with GFAP‐immunoreactive astrocytes (scale bar = 10 μm). (c) Relative fluorescence intensity of TRPM7 normalized to GFAP in astrocytes present in lesions versus astrocytes present in NAWM (Student's *t*‐test, N = 6 for control and 6 for MS). (d) TGF‐β1, not IL‐1α, C1q, or TNF‐α/IFN‐γ, stimulation induces expression of TRPM7 (Student's *t* test, *N* = 3). * *p* < .05, **p < .01, ***p < .001 [Color figure can be viewed at wieyonlinelibrary.com]

TRPM7 was consistently highly expressed in both active, chronic MS lesions compared with control white matter and normal appearing white matter (Figure 1a). No marked differences in cellular expression nor intensity of TRMP7 immunoreactivity was observed comparing control white matter with normal appearing white matter. Antibodies directed against TRMP7 decorated cells with an astrocytic morphology in both active MS lesions as well as the hypocellular centre of chronic lesions (Figure 1a). To confirm the cellular source of TRPM7 expression, we performed co‐localization studies and confirmed that TRPM7 is predominantly expressed by glial fibrillary acidic protein (GFAP)‐positive astrocytes in both active and chronic MS lesions (Figure 1b,c).

Next, we investigated potential regulators of the observed increase in astrocytic TRPM7 expression by exposing primary cortical rat astrocytes to various pro‐inflammatory stimuli known to be abundantly present in MS lesions and reported to have an effect on astrocyte function (Becher et al., 1999; Liddelow et al., 2017; Mizee et al., 2014; van Horssen et al., 2006). TGF‐β, which is prominently expressed in MS lesions (van Horssen et al., 2006), induced a significant and consistent increase in TRPM7 gene expression, whereas exposure to TNF‐α, IFN‐γ, IL‐1α, and complement C1q did not induce mRNA expression of TRPM7 (Figure 1d).

Taken together, our data indicate that TRPM7 expression is markedly increased in reactive astrocytes within active and chronic active MS lesions and that in vitro, TGF‐β1 induces gene expression of TRPM7.

### 3.2 Astrocytes in vitro express functionally active TRPM7

**Figure 2:**
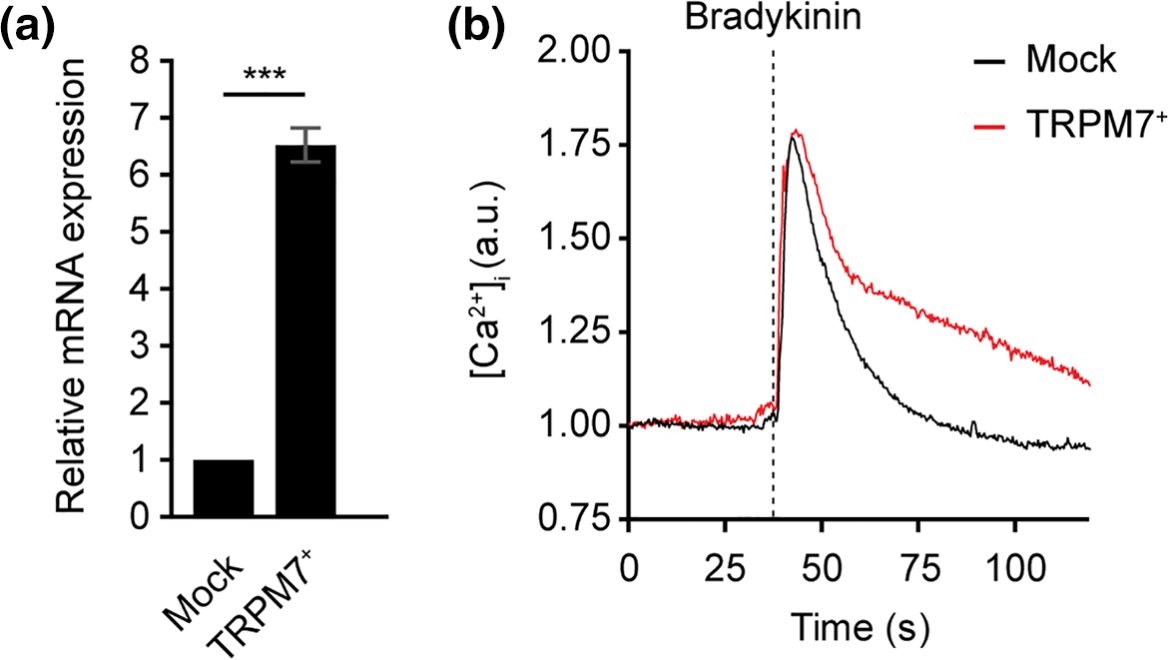
TRPM7 expression in U373/empty vector and U373/TRPM7 cell lines. (a) Gene expression levels of TRPM7 in mock and TRPM7^+^ U373 cells shows increased expression of TRPM7 in TRPM7^+^ U373 astrocytes (Student's *t* test, *N* = 5). (b) Typical time course of bradykinin‐induced Ca^2+^ changes in mock (black line) and TRPM7^+^ (red line) U373 cells. ***p < .001 [Color figure can be viewed at wieyonlinelibrary.com]

U373 astrocytoma cells in which TRPM7 cDNA was stably transduced (TRPM7^+^ astrocytes) were used to further elucidate the role of TRPM7 in astrocytes. TRPM7 overexpression resulted in a six‐fold induction of endogenous TRPM7 levels (Figure 2a). Overexpression of TRPM7 did not alter viability or proliferation as determined by MTS assay (Supporting Information Figure S1). To reveal whether TRPM7 protein is functionally expressed at the cell surface we used a well‐accepted Ca^2+^ imaging approach. We examined the Ca^2+^ influx via TRPM7 by stimulating astrocytes with bradykinin, a PLC‐coupled receptor agonist leading to an IP_3_ mediated Ca^2+^ flux from internal stores. This initial Ca^2+^ flux was followed by a prolonged Ca^2+^ influx, likely via TRPM7, in cells overexpressing TRPM7 compared with mock astrocytes (Figure 2b). These data indicate that TRPM7 is functionally active at the cell surface of TRPM7^+^ astrocytes.

### 3.3 Astrocytic TRPM7 expression inhibits neurite outgrowth

**Figure 3:**
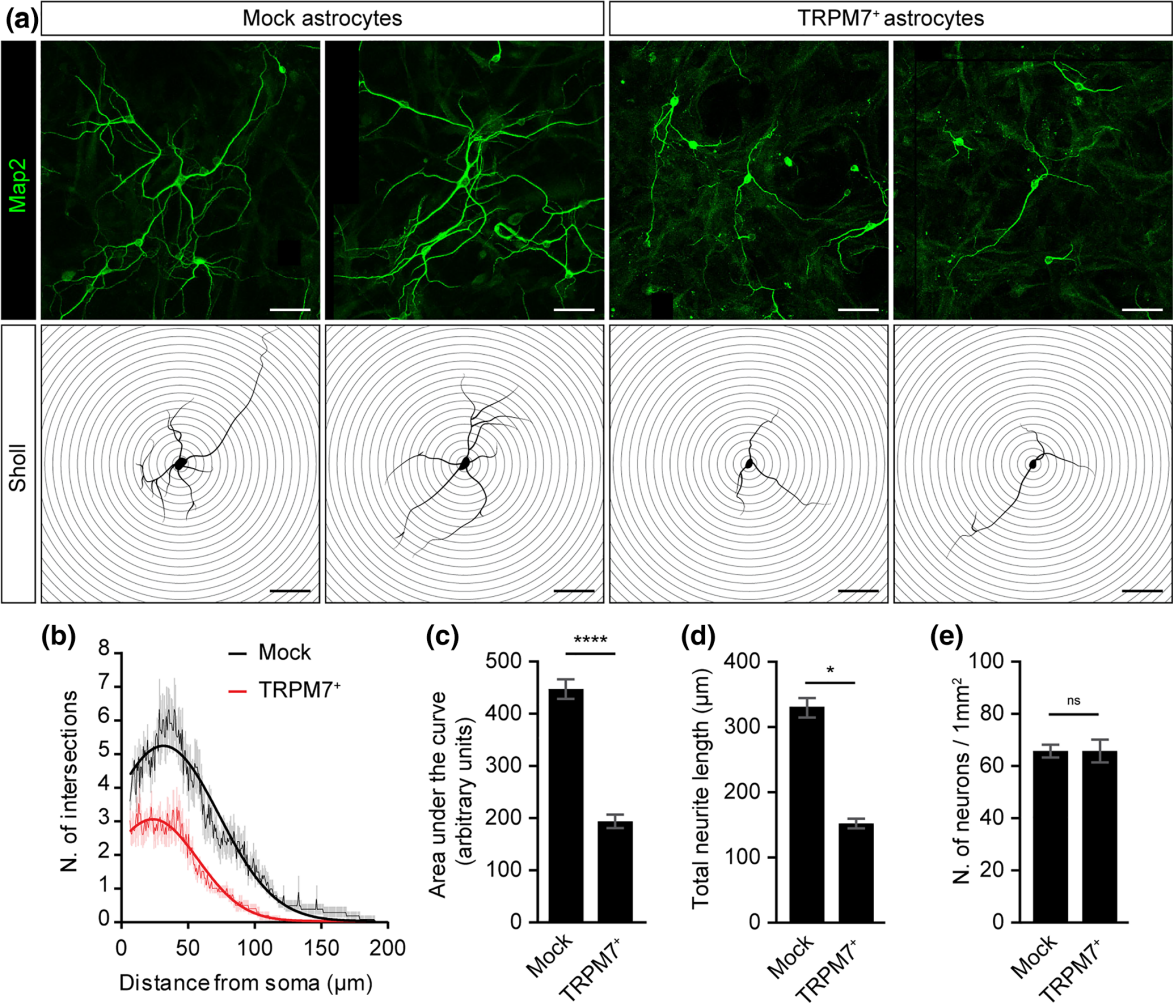
Alterations of neurite morphology of cortical neurons grown on TRPM7 overexpressing astrocytic monolayer. (a) Representative images of MAP2 immunostained cortical neurons on top of mock or TRPM7^+^ astrocyte monolayer (scale bars = 50 μm). (b, c) Quantification by Sholl analysis of neurite complexity revealed impaired neurite complexity of neurons grown on top of TRPM7^+^ astrocytes compared with neurons grown on mock astrocytes. (d) Neurons grown on top of TRPM7^+^ astrocytes show decreased neurite length compared with neurons grown on top of mock astrocytes and (e) Similar total number of neurons (Student's *t* test, *N* = 3 independent experiment, total neurons = 60). *p < .05. ****p < .0001 [Color figure can be viewed at wieyonlinelibrary.com]

Since astrocytes play a central role in neuronal homeostasis and reactive astrocytes hamper neuronal function, we aimed to determine the effect of astrocytic TRPM7 overexpression on neuronal morphology. For that, we cultured primary rat cortical neurons in vitro on monolayers of either mock‐transfected or TRPM7‐overexpressing astrocytes. Using Sholl analysis, we revealed that primary neurons grown on TRPM7‐overexpressing astrocytes, showed a less complex neurite morphology compared with neurons seeded on a mock‐transfected astrocyte monolayer (Figure 3a–c). We observed similar number of neurons in both conditions, indicating that the outgrowth and not the survival of neurons is affected by TRPM7^+^ astrocytes (Figure 3e). Interestingly, no differences in neuronal morphology were found when we prevented physical contact between the astrocytes and neurons. by culturing TRPM7‐overexpressing astrocytes in a Transwell insert with neurons grown on the bottom of the culture plate (data not shown). Taken together, these results demonstrate that overexpression of astrocytic TRPM7 impairs neurite outgrowth, and that a physical interaction is likely needed for the observed effect.

### 3.4 TRPM7 affects neurite outgrowth via affecting CSPG production

**Figure 4:**
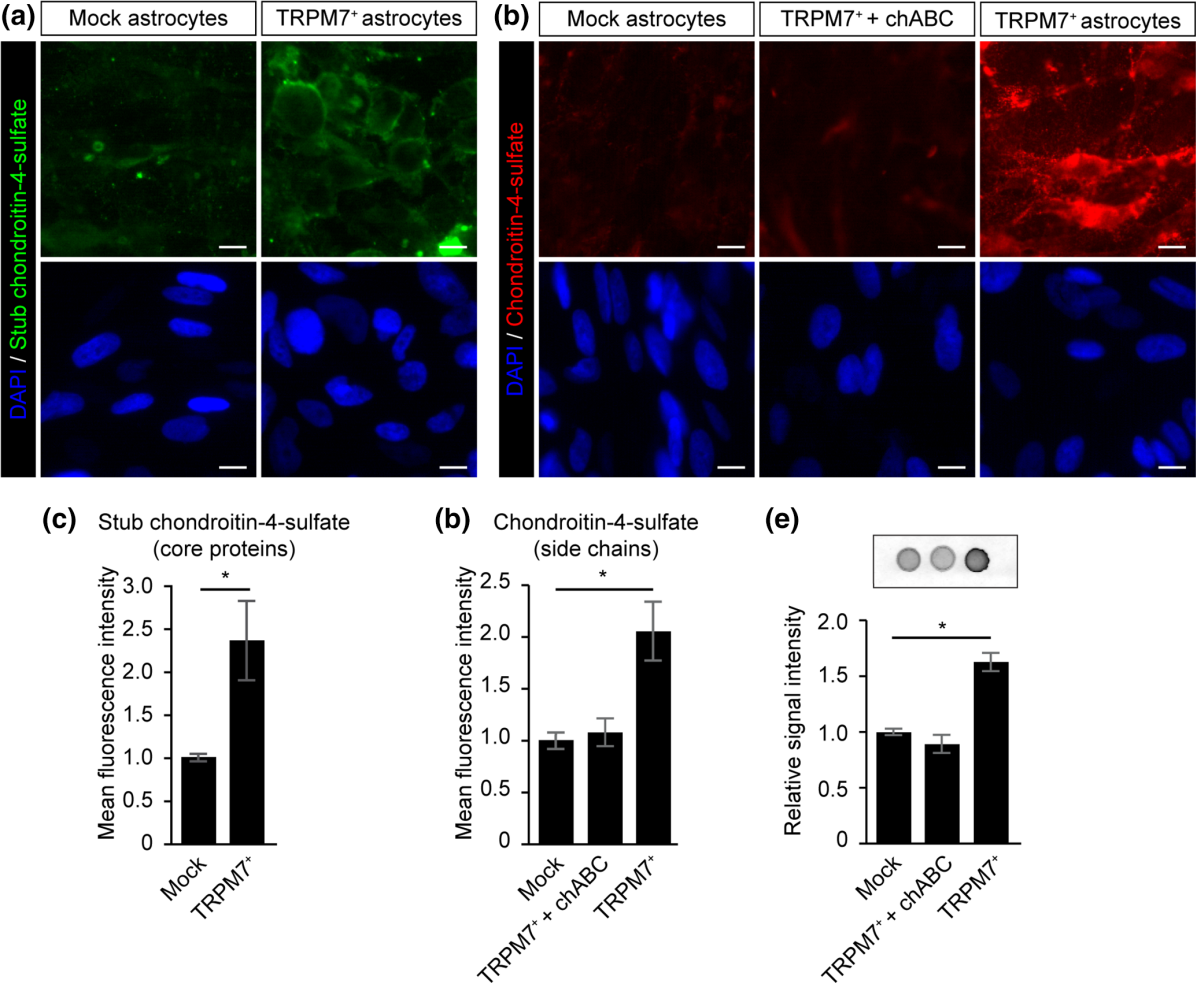
Overexpression of TRPM7 increased CSPG deposition. (a) Representative immunocytochemistry images of stub chondroitin‐4‐sulfate (core proteins) and (b) Chondroitin‐4‐sulfate (side chains) scale bars = 10 μm. (c) Quantifications shows increased intensity of stub chondroitin‐4‐sulfate (core proteins) (Student's *t* test, *N* = 4) and (d) Chondroitin‐4‐sulfate (side chains) (Student's *t* test, *N* = 3) relative to DAPI intensity in TRPM7^+^ astrocytes cells compared with mock. (e) CSPG protein expression in mock astrocytes, chondroitinase ABC‐treated TRPM7^+^ astrocytes, and TRPM7^+^ astrocytes. Quantification showed that CSPG protein production is significantly increased in TRPM7 overexpressing astrocytes compared with mock astrocytes expression highest in (Student's *t* test, *n* = 3) *p < .05 [Color figure can be viewed at wieyonlinelibrary.com]

Impaired neuroregeneration in MS lesions, including disturbed neurite outgrowth, has been mostly attributed to the presence of the gliotic scar, of which CSPGs are a key component (Haylock‐Jacobs, Keough, Lau, & Yong, 2011). Hence, we questioned whether TRPM7 could affect neurite outgrowth via a similar pathway and investigated whether TRPM7 overexpressing astrocytes were involved in the production of CSPGs. Quantification of CSPG production by mock‐ and TRPM7 overexpressing astrocytes was analyzed by means of immunocytochemistry. Both the stub chondroitin‐4‐sulfate core protein as well as chondroitin‐4‐sulfate side chains were enriched in TRPM7 overexpressing astrocytes compared with mock astrocytes (Figure 4a–d). Moreover, enzymatic removal of the chondroitin‐4‐sulfate side chains using chABC resulted a loss of signal (Figure 4b), Dot‐blot analysis confirmed enhanced production of CSPGs by TRPM7‐overexpressing astrocytes (Figure 4e). Immunohistochemical analysis of TRPM7 and CSPG side chain expression revealed that CSPG immunoreactivity is strikingly increased in MS lesions and colocalized with GFAP‐immunopositive astrocytes (Supporting Information Figure S2).

## 4 DISCUSSION

In this study, we showed that TRPM7 is strikingly upregulated in reactive astrocytes in both active and chronic active MS lesions and that TGF‐β1 treatment is able to enhance astrocytic TRPM7 expression in vitro. We demonstrated that astrocytic TRPM7 negatively impacts neuronal outgrowth possibly via increased production of CSPGs. Taken together, we identified TRPM7 as a novel player in MS pathophysiology involved in neuronal outgrowth, which might contribute to impaired neuroregeneration in MS patients.

TRPM7 is weakly expressed in astrocytes within control white matter and normal appearing white matter and strongly expressed in reactive astrocytes within active and chronic active MS lesions. Analysis of publicly available gene expression datasets (GEO accession number GSE38010) revealed that TRPM7 expression was increased in tissue containing active, chronic active or chronic MS lesions in four out of the five cases compared with control cases (Han et al., 2012). TRPM7 is known to be involved in a variety of pathologies, including different cancer types and numerous fibrotic diseases (Middelbeek et al., 2012, 2015; Visser, Middelbeek, van Leeuwen, & Jalink, 2014; Xu et al., 2015). Yet, little is known about a putative role of TRPM7 in neurological diseases. Dysregulation of neuronal TRPM7 activity has been associated with familial Alzheimer's disease, Parkinson's disease and stroke (Landman et al., 2006; Sun et al., 2015).

Overactive neuronal TRPM7 leads to calcium‐induced cytotoxicity during cerebral ischaemia (Aarts et al., 2003; Hermosura et al., 2005) and changes in the function of TRPM7 may either be caused by genetic alterations or changes in regulatory pathways of TRPM7 channel activity (Sun et al., 2015). Enhanced astrocytic TRPM7 protein expression may be the result of the inflammatory environment and the production of TGF‐β1 by activated glia and infiltrated macrophages (Miljković et al., 2011; van Horssen et al., 2006). Exposure of astrocytes to TGF‐β1 induced gene expression of TRPM7 and a recent study described that TGFβ‐induced epithelial‐to‐mesenchymal transition in prostate cancer is mediated via TRPM7 expression (Sun, Schaar, Sukumaran, Dhasarathy, & Singh, 2018). Additionally, fibroblasts exposed to TGF‐β1 promote extracellular matrix formation via upregulation of TRPM7 (Fang et al., 2014). Furthermore, it has previously been shown that treatment of astrocytes with TGF‐β1 induces specific alterations that are consistent with astrogliosis (Cullen, Simon, & LaPlaca, 2007; Logan et al., 1994; Silver & Miller, 2004). Other inducers of a reactive astrocyte phenotype, such as Il‐1α, TNF‐α, and C1q, failed to induce TRPM7 expression in astrocytes in our hands. Interestingly, these mediators are known to induce a so called A1 astrocyte phenotype that is thought be neurotoxic whereas TGF‐β1 on the other hand has been shown to limit the A1 reactive astrocyte phenotype (Liddelow et al., 2017). Based on these observations, we conclude that TRPM7 expression is not associated with the A1 neurotoxic astrocyte phenotype.

To allow in‐depth investigation on the role of astrocytic TRPM7 expression, we generated a U373 astrocyte cell line with functionally active TRPM7. Manipulation of TRPM7 expression in vitro has been proven to be challenging, since strong upregulation, as well as downregulation of TRPM7 induces calcium toxicity and ultimately cell death (Nadler et al., 2001). Furthermore, long‐term incubation with TRPM7 channel blockers also reduces cell viability (Zierler et al., 2011). However, in agreement to our data, it has been shown that a moderate increase in TRPM7 expression does not influence cell viability (Middelbeek et al., 2015).

Using TRPM7^+^ astrocytes as a feeder layer for primary neurons, we observed reduced neuronal outgrowth compared with neurons grown on top of mock‐transfected astrocytes without affecting neuronal number. Despite co‐culture of human U373 astrocytes together with primary rat neurons, we are able to obtain reliable results with this model. It would be interesting to inhibit TRPM7 using Waixenicin‐A, a known specific TRPM7 inhibitor (Zierler et al., 2011), however, due to the ubiquitous expression pattern of TRPM7, it is impossible to selectively target astrocytes.

Reactive astrocytes are known to play a Janus‐faced effect on neuronal outgrowth, by production of neurotrophic factors that facilitates neurite outgrowth and concomitant secretion of neurotoxic factors that hamper neuroregeneration (Landman et al., 2006; Liddelow et al., 2017). We demonstrated that that difference in neurite outgrowth was not mediated via secreted factors suggesting that physical interaction between the TRPM7+ astrocytes and neurons is needed for the observed changes in neuron morphology. Such a mechanism is via production of a glial scar, a physical barrier formed after demyelination to prevent widespread tissue damage. Astrocytes in glial scar tissue produce proteoglycans, such as aggrecan, brevican, neurocan, and versican, which reduce neurite outgrowth (Sobel & Ahmed, 2001). Our immunocytochemical analysis revealed that TRPM7^+^ astrocytes produce abundant levels of CSPGs, well‐known outgrowth inhibitory and permissive molecules (Anderson et al., 2016). Future studies are needed to unravel which specific CSPG species are induced in TRPM7+ astrocytes and the mechanism by which TRPM7 modulates CSPG production.

CSPGs are also known to negatively affect oligodendrocyte maturation and myelination (Keough et al., 2016; Lau et al., 2012). In addition, recent data suggest that CSPGs boost the migratory potential of leukocytes to cross the glia limitans into the CNS, thereby contributing to inflammation (Stephenson et al., 2018). It is therefore possible that enhanced astrocytic expression of TRPM7 contributes to several pathological processes involved in MS lesion development and progression.

Complete prevention or removal of the glial scar causes increased inflammation and tissue damage thereby worsening functional outcome of several neuroinflammatory and neurodegenerative disorders (Anderson et al., 2016; Haroon et al., 2011; Robel, Berninger, & Götz, 2011). Yet, inhibition of CSPGs expressed by astrocytes after CNS insult is linked to improved axonal regeneration after trauma (Cregg et al., 2014; Sharma, Selzer, & Li, 2012). Therefore, a subtle approach, in which only the detrimental effects of the glial scar are targeted, is needed. Interestingly, FTY720, also known as fingolimod, a clinically approved drug to treat MS (Brinkmann et al., 2010) has been shown to be able to block the activity of TRPM7 (Qin et al., 2013). The inhibitory potential of FTY720 is however restricted to the bio‐inactive, non‐phosphorylated form of FTY720. When FTY720 is phosphorylated, it becomes the active compound of fingolimod and loses it inhibitory effect on TRPM7. Future studies are needed to address the question whether part of fingolimods mechanism of action is via inhibiting astrocytic TRPM7 mediated CSPG production. Finally, it will be interesting to study whether astrocyte specific knockout of TRPM7 would alter the clinical symptoms in mice suffering from experimental autoimmune encephalomyelitis (EAE), the animal model for MS. Global deletion of TRPM7 in mice is lethal (Jin et al., 2008), studies investigating the viability of mice with an astrocyte specific knockout of TRPM7 are needed to open up the possibility for an EAE study.

In conclusion, we show that TRPM7 is highly expressed in reactive astrocytes within MS lesions and that enhanced astrocytic TRPM7 levels impair neurite outgrowth by increased production of CSPGs, a key component of the gliotic scar. These findings indicate that astrocytic TRPM7 is a critical regulator of the formation of a gliotic scar and provide a novel mechanism by which reactive astrocytes affect neuronal outgrowth.

## Supporting information


**FIGURE S1** Manipulation of TRPM7 expression does not affect cell viability and proliferation of U737 astrocytoma cells (**a)** Viability was assessed over 4 hr. (**b)** proliferation was assessed over 4 days. Data represents normalized mean extinction at 392 nm

**FIGURE S2** CSPG expression in an chronic active MS lesions (out‐lined by dotted line) colocalized with astrocytic TRPM7 expression. (Scale bar = 100 μm)
