# Supplementary figures and images for: Reactive astrocytes in multiple sclerosis impair neuronal outgrowth through TRPM7‐mediated chondroitin sulfate proteoglycan production
Source: Glia. 2018 Nov 19;67(1):68–77. doi: 10.1002/glia.23526 (PMC6587975; doi:10.1002/glia.23526)

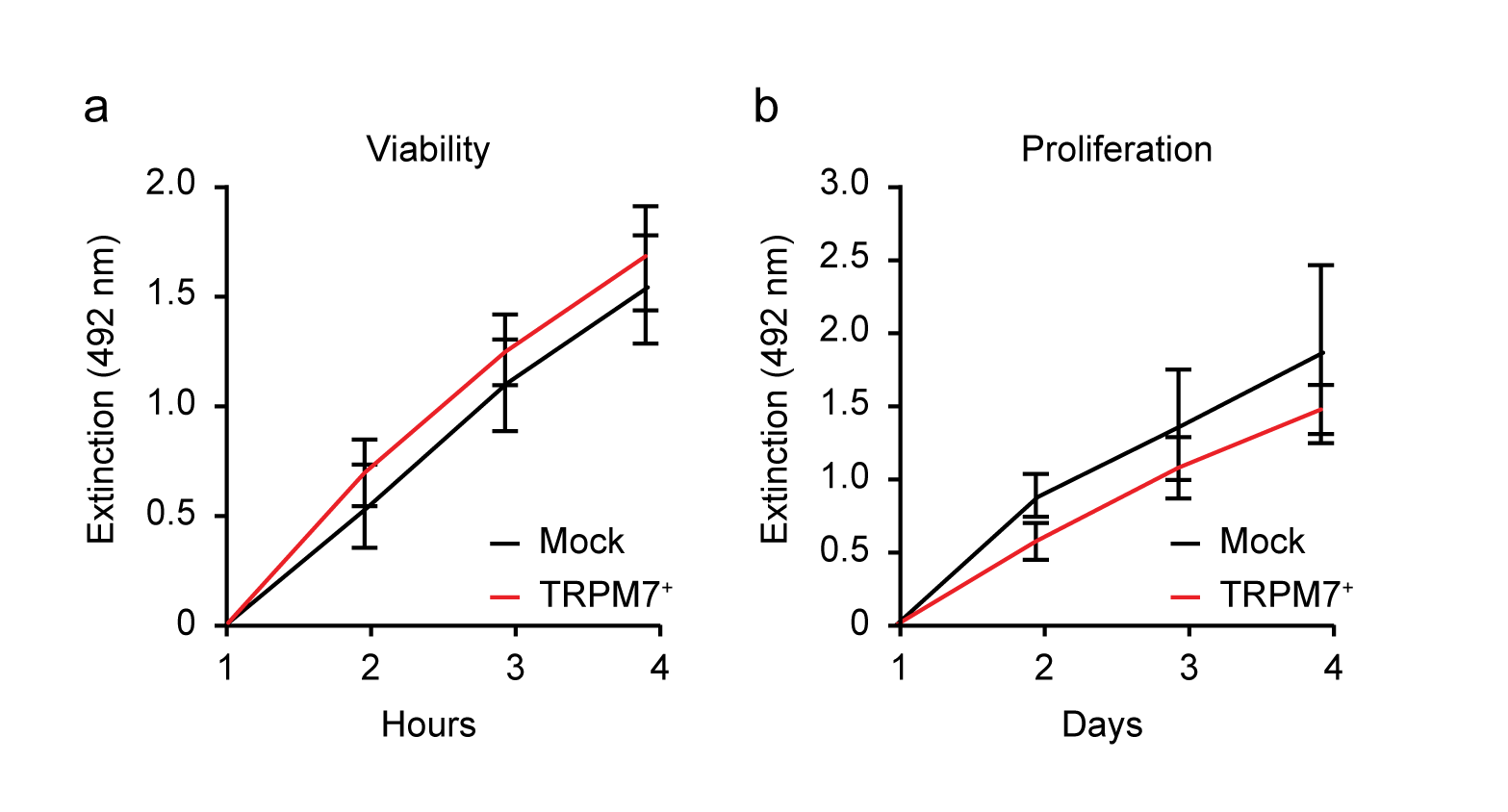

Supplement: Supplementary file 1 — FIGURE S1 Manipulation of TRPM7 expression does not affect cell viability and proliferation of U737 astrocytoma cells (a) Viability was assessed over 4 hr. (b) proliferation was assessed over 4 days. Data represents normalized mean extinction at 392 nm [file GLIA-67-68-s001.zip › glia23526-sup-0001-FigureS1.tif]

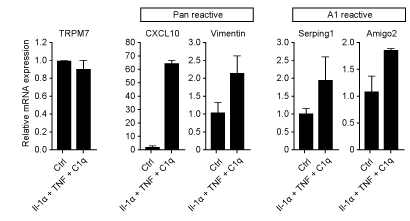

Supplement: Supplementary file 1 — FIGURE S1 Manipulation of TRPM7 expression does not affect cell viability and proliferation of U737 astrocytoma cells (a) Viability was assessed over 4 hr. (b) proliferation was assessed over 4 days. Data represents normalized mean extinction at 392 nm [file GLIA-67-68-s001.zip › Figure10.tif]

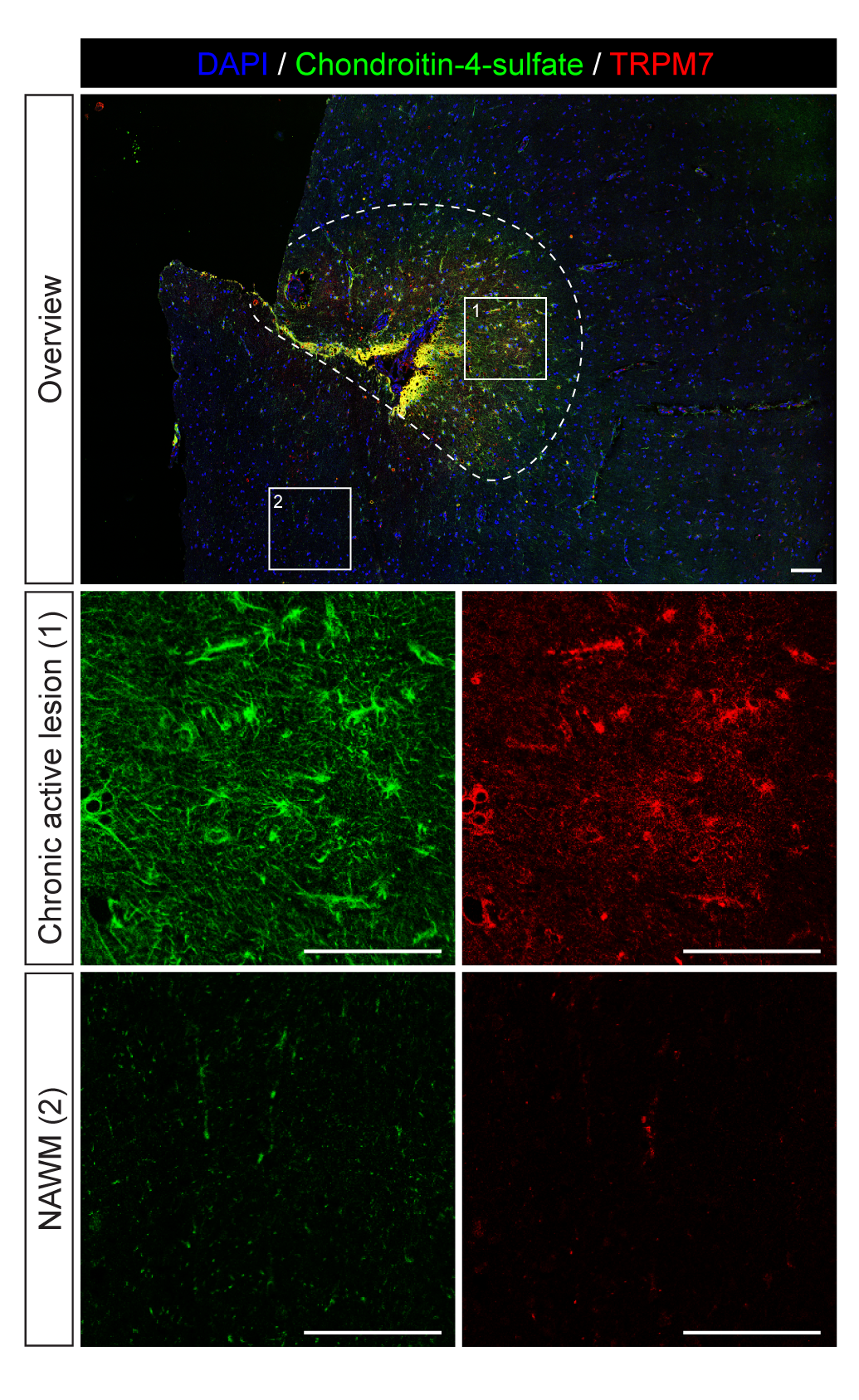

Supplement: Supplementary file 2 — FIGURE S2 CSPG expression in an chronic active MS lesions (out‐lined by dotted line) colocalized with astrocytic TRPM7 expression. (Scale bar = 100 μm) [file GLIA-67-68-s002.tif]
